# Exploring Pesticide Knowledge, Practices, and Health Perceptions Among Farmers in Akkar, Lebanon

**DOI:** 10.3390/ijerph22020260

**Published:** 2025-02-12

**Authors:** Nisreen Hassan Akkouch, Jalal Halwani, Issam Shaarani

**Affiliations:** 1 Water and Environment Science Laboratory, Faculty of Public Health, Doctoral School of Science & Technology, Lebanese University, Tripoli 1300, Lebanon; jhalwani@ul.edu.lb; 2 Faculty of Medicine, Beirut Arab University, Beirut 1107 2809, Lebanon; i.shaarani@bau.edu.lb

**Keywords:** pesticide, knowledge, practice, smallholder farmers, awareness

## Abstract

Background: The unregulated and widespread use of agricultural pesticides in Lebanon has led to critical health and environmental challenges. Small-scale farmers in Akkar, a key agricultural region, are particularly vulnerable due to limited knowledge, unsafe practices, and insufficient access to safety resources. Objectives: This study evaluates the knowledge, practices, and perceived health impacts of pesticide use among small-scale farmers in Akkar. It aims to identify gaps and provide targeted recommendations for interventions that enhance safety, sustainability, and environmental conservation. Methods: A cross-sectional survey involving 151 farmers was conducted from November 2022 to January 2023 using a validated questionnaire. Descriptive statistics, regression analysis, and Tukey’s HSD post hoc tests were used to assess knowledge and practice scores and identify predictors. Results: Farmers demonstrated moderate knowledge (mean score: 0.545) and practices (mean score: 0.607). However, environmental awareness was low, with only 9.3% recognizing water contamination risks. Alarmingly, 37.7% of farmers reported using no protective gear, while 67.5% experienced acute symptoms such as respiratory irritation and skin issues. Education was a significant predictor of knowledge (*p* < 0.01). Conclusions: This study underscores the pressing need for tailored educational programs, accessible protective equipment, stricter regulatory frameworks, and environmental conservation strategies to reduce health risks and promote sustainable pesticide use.

## 1. Introduction

Pesticide use plays a dual role, enhancing crop production while posing significant risks to human health and the environment [1]. These chemicals are instrumental in pest control and yield enhancement [2], but their indiscriminate application can adversely affect soil quality [3], water resources [4], air purity [5], and overall human well-being [6]. Farmers, as the primary handlers of pesticides, are particularly vulnerable, with exposure occurring through skin contact, inhalation, and ingesting contaminated food and water [7,8]. Chronic exposure to pesticides has been linked to various health problems, including carcinogenic, neurological, gastrointestinal, respiratory, dermatological, reproductive, and endocrine-related issues [9]. Recent studies have highlighted the endocrine-disrupting effects of pesticides, emphasizing their potential to disrupt hormonal systems and adversely impact reproductive health, with long-term implications [10].

Globally, governments and organizations such as the Environmental Protection Agency (EPA) and the Food and Drug Administration (FDA) regulate pesticide manufacturing and use. However, policy weaknesses, limited enforcement, and farmers’ ‘lack of knowledge’ in developing countries exacerbate unsafe practices [11]. Acute pesticide toxicity in low- and middle-income countries (LMICs) results in approximately 200,000 deaths annually, often due to insufficient protective measures and poor handling practices [12].

In Lebanon, pesticide use is widespread, particularly in agricultural regions like Akkar and Bekaa, where farmers cultivate fruits, vegetables, and tobacco [13]. Nevertheless, the country encounters significant challenges in pesticide regulation, both during and after the registration process. According to Abu Zeid et al., Lebanon’s pesticide registration system relies on international reference countries, including the EU, USA, and Japan, without conducting local residue trials. Additionally, inadequate life cycle management at the post-registration stage intensifies the issue, leading to pesticide misuse and increased exposure to harmful residues for both farmers and consumers [14]. These challenges are further aggravated by weak regulatory enforcement and the persistent use of banned or outdated chemical products [15]. Studies report that nearly 49% of Lebanese farmers engage in unsafe pesticide practices, resulting in illegal residues in environmental matrices [16] and food products [17,18]. The ongoing economic crisis and Syrian refugee influx have further strained resources, reducing farmers’ ability to adopt safer agricultural practices [19].

Despite the widespread use of pesticides in Akkar, research in this region has predominantly focused on environmental assessments, such as water contamination and pesticide residues [20,21]. No studies have comprehensively evaluated farmers’ knowledge, attitudes, and practices (KAPs) regarding pesticide safety, nor have there been interventional studies to raise awareness among farmers and the broader community. This lack of research is significant, as such studies are essential for designing tailored strategies to address the unsafe use of pesticides and mitigate associated health and environmental risks.

The Akkar Valley, Lebanon’s second-largest agricultural region after the Bekaa Valley, is a vital hub for farming but faces unique socioeconomic and environmental challenges. These include high poverty rates, limited infrastructure, and low literacy levels among farmers, which exacerbate their vulnerability to pesticide-related health and environmental risks [13,22], Despite evidence of significant exposure levels in this region [21], no prior studies have explored the acute health symptoms experienced by farmers or evaluated their knowledge, attitudes, and practices (KAPs) regarding pesticide safety.

KAP studies are instrumental in identifying knowledge gaps, misconceptions, and unsafe behaviors that hinder the adoption of preventive measures [23]. They provide essential baseline data for designing locally relevant interventions [24]. For example, a recent study assessing Lebanese farmers’ understanding of pesticide pictograms revealed significant gaps in interpreting these symbols, underscoring the need for improved training and communication methods tailored to local contexts [25]. In Akkar, where agricultural activity is significant and pesticide-related risks are high, this study addresses a critical gap by evaluating farmers’ knowledge and practices regarding pesticide use.

This study specifically assesses farmers’ understanding of the health and environmental impacts of plant protection products (PPPs), their adherence to PPE usage, and their pesticide storage and disposal practices. It also examines acute health effects, calculates knowledge and practice scores, and explores factors shaping attitudes toward pesticide safety. These findings aim to inform policymakers, non-governmental organizations (NGOs), and agricultural stakeholders, offering actionable recommendations for promoting safer and more sustainable farming practices in Lebanon.

## 2. Methodology

### 2.1. Study Design

A cross-sectional study was conducted between November 2022 and January 2023 to assess pesticide knowledge, practices, and perceived health impacts among smallholder farmers in ten coastal villages within the Akkar Valley. This design allowed us to capture a snapshot of pesticide-related factors at a specific point in time. Participants were selected using convenience sampling, targeting farmers actively involved in pesticide handling who were willing to participate. This method facilitated efficient data collection, given the available resources and time constraints. Ethical approval was obtained from the Doctoral School of Science and Technology at the Lebanese University (IRB number CE-EDST-6-2022), ensuring the protection of participant rights, confidentiality, and informed consent.

### 2.2. Study Areas

Akkar is Lebanon’s second-largest agricultural area after the Bekaa Valley, characterized by mountainous and plain municipalities. The primary crops grown in Akkar include wheat, fruits, vegetables, olives, and tobacco. This region faces significant socioeconomic challenges, including high illiteracy rates and inadequate infrastructure, such as the absence of public potable water services, leading residents to rely on well water for daily needs, including cooking, drinking, and irrigation [13].

**Figure 1:**
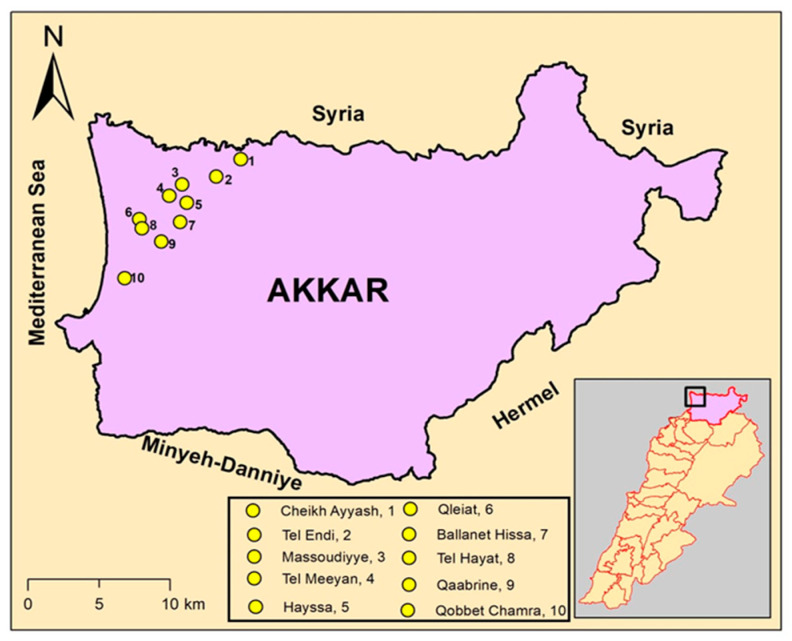
Map of Akkar (34.5303° N, 36.1612° E) showing the ten selected villages and the number of participants from each village.

For this study, ten coastal villages were selected based on shared characteristics, including similar altitude levels, crop types, and the presence of both open fields and greenhouse agriculture. Figure 1 illustrates the map of Akkar, highlighting the selected villages and the number of participants recruited from each village.

### 2.3. Questionnaire Development

The questionnaire was designed based on a comprehensive review of validated knowledge, attitude, and practice (KAP) studies [12,26,27,28]. It was reviewed and refined by an expert team comprising a professor specializing in Environment and Health, an epidemiologist, and a family physician with experience in research methodology, ensuring content validity and relevance.

The final questionnaire consisted of 25 questions distributed across four sections:

1. Demographics: Six questions were included to gather information about village name, age, gender, educational level, years of farming experience, and participation in training or workshops on agricultural safety.
2. Knowledge of Health and Environmental Impacts: Eleven questions assessed farmers’ awareness of pesticide exposure routes (e.g., through skin contact, inhalation, and ingestion) and their understanding of the risks associated with unsafe pesticide use. Topics included the effects of exceeding recommended doses, frequent spraying, and the potential for pesticides to harm human health, pollute water resources, and negatively impact beneficial pests like bees.
3. Pesticide Use and Practices: Seven questions examined farmers’ adherence to safety practices, including the use of personal protective equipment (PPE), following dosage instructions, precautions taken after spraying (e.g., washing hands and face or taking a shower), storage practices for pesticides, and whether pesticides were applied during unsuitable conditions such as windy weather.
4. Perceived Health Impacts: One multiple-choice question was asked with 20 options, allowing participants to report acute symptoms or health effects experienced due to pesticide exposure.

### 2.4. Refinement Through Pilot Study

A pilot study was conducted with 20 farmers from two villages to test the questionnaire’s clarity, relevance, and structure. Feedback from the pilot led to improvements in question phrasing and the addition of response options (e.g., “I don’t know” for knowledge questions). The pilot data were excluded from the final analysis to maintain dataset integrity, as changes were made to the questionnaire after the pilot phase.

### 2.5. Validation and Reliability

The questionnaire’s reliability was assessed using Cronbach’s alpha coefficients [29], which yielded values of 0.70 for knowledge-related questions and 0.72 for practice-related questions, indicating acceptable internal consistency. Additionally, a test–retest method was performed with three farmers, who completed the questionnaire twice, one week apart [30]. The responses demonstrated consistent results, confirming the reliability of the instrument.

### 2.6. Data Collection

The finalized questionnaire was translated into Arabic, the official language of Lebanon, to ensure comprehension. It was administered face-to-face by trained surveyors proficient in Arabic. These surveyors, experienced in similar research, received comprehensive training on interview techniques, ethical considerations, informed consent, and confidentiality. Informed consent was obtained from all participants, with fingerprinting used as a substitute for signatures for illiterate farmers.

To ensure anonymity and data quality, no identifying information was collected, and responses were recorded directly using Google Forms, minimizing transcription errors. No missing data were observed across the responses, as the questionnaire was administered face-to-face, ensuring complete answers from all participants. Continuous supervision during data collection allowed the team to address discrepancies or issues promptly.

### 2.7. Statistical Analysis

The collected data were coded and analyzed using the Statistical Package for Social Sciences (SPSS) software, version 25. Descriptive statistics, including frequencies and percentages, were used to summarize the results. Cronbach’s alpha coefficients were calculated to assess the internal consistency of the questionnaire. Knowledge and practice scores were calculated on a scale of 0–1, where a score of 0 indicated all incorrect answers and 1 indicated all correct answers.

Before conducting parametric tests, assumptions of normality and homogeneity of variance were assessed. Normality was tested using the Shapiro–Wilk test [31], and homogeneity of variance was assessed using Levene’s test [32]. For both tests, a *p*-value > 0.05 indicated that the assumptions for parametric analysis were satisfied. Based on these results, independent sample *t*-tests and an analysis of variance (ANOVA) were used to compare mean scores across the demographic groups.

A *p*-value of ≤0.05 was considered statistically significant. For post hoc analysis, Tukey’s Honest Significant Difference (HSD) test was applied to identify significant differences between groups where applicable.

## 3. Results

### 3.1. Demographics: Characteristics and Profile of the Farmers

Table 1 displays the key characteristics of the interviewed farmers, including their gender, age, general education, and agricultural training status. A total of 151 participants participated in this study, with 90.7% being men and 9.3% being women. Around 70.9% were over 40 years old. Most of the farmers attended primary or middle school, with 39.1% and 37.7%, respectively. Almost 69% of the participants had not participated in any training or workshop related to good agricultural practices (GAPs) and pesticide safety. Most participants (47.6%) had more than 20 years of experience.

### 3.2. Participants’ Knowledge Regarding Plant Protection Products

Overall, the farmers’ knowledge about pesticides was moderate (Table 2). A significant portion of our participants recognized the pesticide routes of exposure. The majority of farmers (78.1%) were aware that not wearing goggles while preparing pesticides could lead to eye splashes and irritation. However, a high percentage of farmers (60.6% and 63.6%) believed that applying more than the recommended amount of pesticides and increasing the frequency of crop spraying could boost yields and provide protection. It is concerning that only 9.3% of farmers were aware that the use of pesticides in agriculture could contaminate water resources, and only half of the participants believed that pesticide exposure could harm humans.

### 3.3. Participants’ Practice Regarding Pesticide Handling

According to the findings, 51.7% of farmers follow the exact dosage recommended by the agricultural veterinarian, while 47.7% increase the pesticide concentration for more effective pest control (Table 3). Interestingly, only 4% of farmers read and follow the pesticide label; the remaining farmers rely on their personal experience or advice from family members and pesticide retailers. Regarding protective gear, 37.7% of the farmers do not use any protective gear. Of the remaining participants, 13.9% use gloves, 2% use goggles, and 45% use facial masks.

### 3.4. Participants’ Knowledge and Practice Scores

The mean practice score was 0.607, with a standard deviation of 0.135 (Table 4). When comparing the mean practice scores between personal characteristics, no statistically significant differences were observed in the practice scores regarding gender, age, education, experience, and training. Farmers exhibited moderate knowledge levels (mean score: 0.545, SD: 0.198), revealing substantial room for improvement in safe pesticide use practices. When comparing the mean knowledge score between the personal characteristics, the associated statistical analysis (*t*-test) yielded non-significant *p*-values for age, gender, and experience (Table 5). In regard to educational level, the participants were divided into four education groups: “Illiterate”, “Primary School”, “Middle school”, and “Secondary and more”. The average scores were 0.437, 0.535, 0.563, and 0.682, respectively. The ANOVA analysis showed a significant score difference (*p* = 0.003). Two subgroups with extremes were found: Illiterate, Primary School, and Middle School in one, and Middle School with Secondary and more in the other (Table 5).

### 3.5. The Prevalence of Acute Symptoms Recorded by the Participants

**Figure 2:**
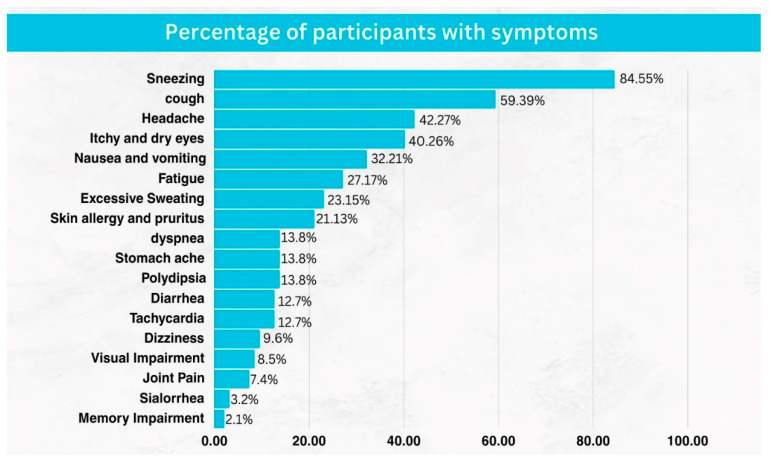
Farmers’ self-perceived acute toxicity symptoms (N = 102).

Our research shows that out of the 151 farmers we interviewed, only 32.5% (49 people) did not experience any symptoms after preparing and spraying pesticides. The most commonly reported symptom was sneezing, affecting 84.55% of participants, while the least reported symptom was memory impairment, observed in only 2.1%. Other frequently reported symptoms included coughing (59%), headaches (42%), itchy and dry eyes (40%), nausea or vomiting (32%), and fatigue (27%). Figure 2 illustrates the prevalence of acute symptoms among the 102 participants who experienced them, ranked from most to least common.

## 4. Discussion

The knowledge, practices, and health impacts of plant protection products among farmers in Lebanon have not been thoroughly studied. Identifying these gaps is crucial for policymakers and stakeholders in agriculture and public health. Our study focused on farmers in Akkar, northern Lebanon. Most participants were middle-aged men, reflecting a common trend in many Arab nations where men dominate agricultural activities. Younger individuals showed less interest in agriculture, likely due to aspirations for higher education and urban lifestyles [39].

Most surveyed farmers had basic education and extensive agricultural experience but lacked formal training in safe pesticide handling. This reliance on intergenerational knowledge transfer can hinder the adoption of modern agricultural technologies [40]. Studies indicate that less formal education correlates with poorer pesticide handling practices, and older, experienced farmers often neglect safety precautions [27,37,41]. Tailored educational interventions are essential to improve farmers’ understanding of safe agricultural practices [42].

Despite reported awareness of pesticide exposure routes, PPE usage was limited, with 37.7% not using any protective gear. Facial masks were the most common PPE (45%), followed by gloves (13.9%) and goggles (2%). This insufficient use of PPE exposes farmers to acute and chronic health risks, as evidenced by a high prevalence of acute symptoms. Similar findings on PPE compliance were reported in Kuwait [26], Indonesia [43], and Greece [33], contrasting with better compliance in Palestine [27]. Proper PPE significantly reduces pesticide exposure and health impacts [44].

A substantial proportion of farmers (61.6%) in our study believed that exceeding recommended pesticide doses and increasing spraying frequency (63.6%) would improve crop yield, indicating significant misconceptions. Similarly, a study by Maddah et al. found that 45% of participants held the same belief, highlighting a widespread issue across Lebanese farming communities [45]. This misuse can lead to health impacts, pest resistance, harm to beneficial organisms, and excessive residue on crops [1]. The continued use of outdated pesticides in Lebanon further complicates the issue [15].

Environmental awareness was low, with only 9.3% recognizing the potential for water contamination and less than half aware of broader environmental impacts. Pesticides, when misused, can contaminate soil, groundwater, and surface water, leading to long-term environmental degradation [1]. Akkar’s heavy reliance on well water for irrigation and domestic use heightens the risk of human exposure to pesticide residues. Studies have shown that improper pesticide disposal, including unsafe rinsing and discarding of containers, contributes significantly to this contamination [36,46].

Additionally, pesticides can disrupt the ecological balance by harming beneficial organisms like pollinators and natural pest predators, leading to a decline in biodiversity [47]. Documented water contamination in Akkar [20] underscores the pressing need for targeted education on sustainable pesticide practices and proper waste management to protect environmental health. Sustainable strategies, such as using only the required quantities, implementing integrated pest management (IPM), and adopting safe disposal methods, are critical for reducing the environmental footprint of pesticides in the region [46].

Post-spraying hygiene practices were generally good, with most farmers storing pesticides safely. However, the unsafe disposal of empty containers was common, posing significant environmental and health risks [48]. Effective pesticide waste management is crucial, involving purchasing only necessary quantities, proper rinsing, and safe disposal [49].

No significant differences in knowledge and practice scores were found based on gender (*p* = 0.998 for practice, *p* = 0.157 for knowledge), age (*p* = 0.619 for practice, *p* = 0.189 for knowledge), years of experience (*p* = 0.893 for practice, *p* = 0.323 for knowledge), or training (*p* = 0.862 for practice, *p* = 0.036 for knowledge). However, higher education correlated with better knowledge (*p* = 0.003), underscoring the need for targeted educational interventions.

The effectiveness of educational interventions in improving farmers’ knowledge, attitudes, and practices regarding pesticide use has been well documented. For example, Maddah et al. demonstrated that targeted training can address common misconceptions, such as the belief that higher pesticide doses improve crop protection, while also encouraging better compliance with PPE and hygiene practices [45]. While our study did not include an intervention, the shared challenges identified across Lebanese regions underscore the need for educational programs to enhance farmers’ KAPs and promote safer pesticide practices.

The prevalence of acute symptoms was high, with respiratory issues, headaches, and eye irritation being common. These findings align with those from Kuwait [26] reinforcing the urgent need for preventive measures. Pesticide exposure is linked to respiratory [50] and neurological diseases [38] and endocrine disruption [34]. Comprehensive studies and tailored training programs are essential to mitigate these risks and enhance farmer safety.

In conclusion, this study and similar research underscore the urgent need for comprehensive, community-based educational programs to promote safe pesticide use in Lebanon. Policymakers should prioritize accessible training, subsidized PPE, stricter regulatory enforcement, and environmental conservation strategies to reduce health risks and environmental contamination while ensuring sustainable agricultural practices.

## 5. Limitations

This study has several limitations that should be considered when interpreting the findings. First, the relatively small sample size (151 farmers) may limit the generalizability of the results to the broader farming population in Akkar and other regions. However, efforts were made to include farmers from ten villages with diverse backgrounds to enhance the representativeness of the sample.

Second, the reliance on self-reported data may introduce biases, such as recall bias or social desirability bias, which could affect the accuracy of responses regarding pesticide use and health impacts. To mitigate this, we carefully constructed neutral questions to avoid influencing participants’ answers.

Third, while farmers reported acute symptoms potentially related to pesticide exposure, these symptoms cannot be definitively attributed to pesticides alone, as they may also result from factors such as heat, fatigue, or pre-existing health conditions.

Despite these limitations, this study provides critical baseline insights into pesticide handling practices and associated health risks among farmers in Akkar. These foundational data highlight the need for further research, including larger, probabilistic studies and the use of observational or biological measures to validate self-reported findings.

## 6. Conclusions and Recommendations

In Lebanon, the unsafe use of plant protection products poses significant risks to public health and environmental sustainability. This study highlights critical gaps in pesticide knowledge, practices, and environmental awareness among farmers in Akkar, emphasizing the urgent need for intervention. Nearly half of the farmers demonstrated limited knowledge about the health and environmental risks of pesticide use, relying primarily on personal experience rather than professional guidance. The insufficient use of personal protective equipment (PPE) and improper disposal of pesticide containers further exacerbate these risks.

Key recommendations include the following:

1. Policy Reforms:
  ○ Implement stringent regulations on pesticide importation and sales.
  ○ Penalize the illegal trade of prohibited products and promote the use of environmentally safer alternatives.
  ○ Develop and enforce guidelines for the proper disposal of used pesticide containers, with oversight from the Ministry of Agriculture.
2. Educational Interventions:
  ○ Organize targeted training programs focused on safe pesticide handling, reading and interpreting labels, and understanding environmental impacts.
  ○ Provide educational materials in Arabic with clear instructions, larger fonts, and visual aids for low-literacy participants.
  ○ Conduct workshops and focus groups in collaboration with municipalities to enhance farmer participation and community engagement.
3. Resource Accessibility:
  ○ Improve access to affordable PPE and incentivize its usage through subsidies or discounts.
  ○ Establish local agricultural offices to provide expert guidance and support for integrated pest management (IPM).
  ○ Offer financial support, such as long-term loans, to promote soil restoration and sustainable agricultural practices.
4. Research and Monitoring:
  ○ Conduct longitudinal studies to assess long-term effects of educational interventions on farmers’ knowledge, practices, and health.
  ○ Monitor pesticide residues in crops and water sources to ensure compliance with safety standards and protect environmental integrity.

By implementing these strategies, Lebanon can address the challenges of unsafe pesticide use, safeguard public health, and promote sustainable agricultural practices. Collaboration among policymakers, local communities, and agricultural stakeholders is essential to create a safer and more sustainable farming environment.

## Figures and Tables

**Table 1:** Demographic and agricultural characteristics of the study participants (N = 151).

Gender	Frequency	Percent
Male	137	90.7
Female	14	9.3
Age Range	Frequency	Percent
Less than 40 years	44	29.1
40 years or more	107	70.9
Educational level	Frequency	Percent
Illiterate	21	13.9
Primary School	59	39.1
Middle school	57	37.7
Secondary and more	14	9.3
Experience	Frequency	Percent
10 years or less	39	25.8
[5,12,13,26,29,32,33,34,35,36]	40	26.5
[2,3,8,9,10,15,30,37,38,39]	44	29.1
more than 30 years	28	18.5
Did you receive any training/workshop or technical support on safe pesticide handling techniques?	Frequency	Percent
No	104	68.9
Yes	47	31.1
Total	151	100.0

**Table 2:** Knowledge of the participants regarding pesticide use (N = 151).

Questions	Answers	Frequency (n)	Percent (%)
Do you think pesticides enter the human body through skin contact?	No	60	39.7
	I don’t know	15	9.9
	Yes (correct answer)	76	50.3
Do you think pesticides enter the human body through the respiratory system (inhalation)?	No	25	16.6
	I don’t know	2	1.3
	Yes (correct answer)	124	82.1
Do you think eating while handling pesticides will cause accidental oral exposure?	No	42	27.8
	I don’t know	2	1.3
	Yes (correct answer)	107	70.9
Do you think drinking while handling pesticides will cause accidental oral exposure?	No	45	29.8
	I don’t know (No answer)	16	10.6
	Yes (correct answer)	90	59.6
Do you think smoking while handling pesticides will cause accidental oral exposure?	No	32	21.2
	I don’t know (No answer)	18	11.9
	Yes (correct answer)	101	66.9
Would preparing or spraying pesticides without wearing goggles cause eye irritation?	No	26	17.2
	I don’t know	7	4.6
	Yes (correct answer)	118	78.1
Does exceeding the required dose of pesticides help in increasing the yield?	No (correct answer)	57	37.7
	I don’t know	1	0.7
	Yes	93	61.6
Will spraying pesticides more frequently provide better yield protection?	No (correct answer)	53	35.1
	I don’t know	2	1.3
	Yes	96	63.6
Does pesticide use pollute water resources?	No	124	82.1
	I don’t know	13	8.6
	Yes (correct answer)	14	9.3
Does pesticide use negatively affect the environment and kill good pests like bees?	No	40	26.5
	I don’t know	18	11.9
	Yes (correct answer)	93	61.6
Do you think that pesticide exposure affects human health?	No	70	46.4
	I don’t know	8	5.3
	Yes (correct answer)	73	48.3

**Table 3:** Pesticide use practices by the study participants (N = 151).

Questions	Answers	Frequency (n)	Percent (%)
Do you follow the exact instructions (the exact dosage)?	Always (recommended answer)	78	51.7
	Sometimes	72	47.7
	Never	1	0.7
What kind of protection do you use in most cases to protect yourself? Select all the used gear.	Face covered with a piece of cloth	22	14.6
	Facial mask	68	45.0
	Gloves	21	13.9
	Goggles/Glasses	3	2.0
	Hat	4	2.6
	Coverall	0	0.0
	Nothing	57	37.7
The number of protection gear? (Based on the previous question)	Nothing	59	39.1
	One kind	70	46.4
	Two kinds (correct answer)	18	11.9
	Three kinds (correct answer)	4	2.6
What precautions do you take after you finish spraying?	Take a shower at the end of the day	107	70.9
	Take a shower directly (recommended answer)	44	29.1
Wash your face and hands.	No	14	9.3
	Yes (correct answer)	137	90.7
Where do you store pesticide products, sprayers, and mixing materials?	Regular kitchen cabinet	1	0.7
	Specific locked room, shed, or cabinet designed for agriculture materials (recommended answer)	131	86.8
	At home	6	4.0
	Buy needed quantity	11	7.3
	No answer	2	1.3
Do you spray pesticides during windy weather?	Sometimes	11	7.3
	Never (recommended answer)	138	91.4
	No answer	2	1.3
Total		151	100.0

**Table 4:** Scores of knowledge and practices.

Scores	Mean of Knowledge	Standard Deviation “SD”	Minimum	Maximum
Knowledge	0.545	0.198	0	1
Practices	0.607	0.135	0	1

**Table 5:** Association between scores and demographic characteristics.

Variable	N	Mean	Standard Deviation “SD”	F or T Statistics	*p*-Value	Subset 1 *	Subset 2 *
Knowledge score (total)	151	0.545	0.198				
Gender							
Male	137	0.553	0.204	2.021	0.157		
Female	14	0.474	0.114				
Age							
Less than 40 years	44	0.579	0.208	1.738	0.189		
40 years or more	107	0.532	0.193				
Education							
Illiterate	21	0.437	0.169			0.4372	
Primary School	59	0.535	0.199	4.857	0.003	0.5347	
Middle school	57	0.563	0.194			0.5630	0.5630
Secondary and more	14	0.682	0.170				0.6818
Experience							
10 years or less	39	0.578	0.210				
[5,12,13,26,29,32,33,34,35,36]	40	0.568	0.196	1.171	0.323		
[2,3,8,9,10,15,30,37,38,39]	44	0.506	0.194				
More than 30 years	28	0.529	0.187				
Training							
No	104	0.568	0.182	4.502	0.036		
Yes	47	0.495	0.223				

Subsets from Tukey’s HSD post hoc test: Subset 1 (“Illiterate”, “Primary School”, “Middle School”) and Subset 2 (“Middle School”, “Secondary and More”). Groups in the same subset are not significantly different. The star (*) indicates that subset 1 and subset 2 are significantly different from each other.

## Data Availability

The data supporting this study’s findings are available upon request from the corresponding author. Please contact Nisreen Akkouch at nisreenakkouch@gmail.com for access to the data.
